# Probiotics ameliorate endocrine disorders via modulating inflammatory pathways: a systematic review

**DOI:** 10.1186/s12263-024-00743-8

**Published:** 2024-03-19

**Authors:** Marzieh Nemati, Bahareh Ebrahimi, Nima Montazeri-Najafabady

**Affiliations:** 1 Endocrinology and Metabolism Research Center, Shiraz University of Medical Sciences, Shiraz, Iran; 2 Geriatric Research Center, Shiraz University of Medical Sciences, Shiraz, Iran; 3 Biotechnology Research Center, Shiraz University of Medical Sciences, Shiraz, Iran

**Keywords:** Probiotics, *Lactobacillus*, Diabetes, Endocrine disorders, Inflammatory pathways

## Abstract

Probiotics has offered a new prospect to treat and manage a variety of endocrine disorders such as obesity, diabetes, non- alcoholic fatty liver disease and metabolic syndrome. The precise mechanisms by which probiotics exert their beneficial effects on endocrine disorders and its associated problems are still indecisive. It seems that regulating the immune system and suppressing pro-inflammatory pathways like tumor necrosis factor-α and interleukin-6 or triggering anti-inflammatory pathways like interleukin-4 and 10 may be one of the potential mechanisms in the managing of endocrine disorders. In this systematic review, we hypothesized that various probiotic strains (*Lactobacillus*, *Biofidiobacteria*, *Streptococcus*, *Entrococcus*, *Clostridium*, and *Bacillus*) alone or in combination with each other could manage endocrine disorders via modulating inflammatory pathways such as suppressing pro-inflammatory cytokines (IL-6, IL-12, TNF-α, TNF-β, NFκB, and MCP-1), stimulating anti-inflammatory cytokines (IL-4,IL-6, IL-22, IL-23, IL-33, and TGF-β) and maintaining other factors like C-reactive protein, Toll like receptors, LPS, and NK cells. Data source this search was performed in PubMed and Scopus. Both human and animal studies were included. Among more than 15,000 papers, 25 studies were identified as eligible for more assessments. Quality assessment of the studies was cheeked by two researchers independently by title and abstract screening, then article which have inclusion criteria were included, and data retrieved from the included full text studies as the authors had originally reported. Results specified that *Lactobacillus* has been the most widely used probiotic as well as which one exhibiting the extend of the therapeutic effects on endocrine disorders, especially obesity by modulating immune responses. Also, most studies have revealed that probiotics through suppressing pro-inflammatory pathways specially via reducing levels TNF-α cytokine exhibited protective or beneficial effects on endocrine diseases particularly obesity as well as through decreasing level of IL-6 induced therapeutic effects in diabetes. This systematic review suggests that probiotics could ameliorate endocrine disorders via their immunomodulatory effects.

## Introduction

The high frequency of endocrine dysfunctions such as obesity (14%), diabetes (6.1%), metabolic syndrome (31.4%) and non-alcoholic fatty liver disease (NAFLD) (32%) is a worldwide health issue that entails huge healthcare costs [1–4]. The quick progression of these disorders is accompanying with the alteration in the interconnecting between environmental factors, genetic and epigenetic factors [5]. The endocrine system is a multifaceted network that comprises of various glands throughout the body and by using chemical messenger molecules called hormones regulates many functions inside the body. There is a cross-link between NAFLD, obesity, diabetes, and metabolic syndrome, which can cause or exacerbate each other, and lead to many disorders throughout the body [6].

Therefore, their treatment is needed for preventing or mitigating complications caused by hormone imbalance such as high blood glucose in diabetics due to insufficient insulin secretion or response [7] and insulin resistance in people with obesity [8, 9] or NAFLD [10]. Several treatment approach are accessible including non-pharmacological therapy such as changing lifestyle, diet, and physical activity, and pharmacological therapy [11].

In spite of the application of pharmaceutical products in the treatment of endocrine disorders, they easily prompt a few serious side effects. Recently, more considerations are paid to use the other treatment options with less side effects and more compatibility like probiotic application for treating various endocrine diseases.

Probiotics are living microorganisms that their extraordinary and protective effects on various tissues have been reported. For example, studies have shown that administering adequate doses of probiotics protects the heart against damages [12–14], increases metabolisms [15], modulates immune system function [13, 16] participates in gastrointestinal health [16, 17] and may have beneficial effects on mood as well [18]. Also, it has been indicated that gut microbiota or probiotics have various important functions of the host in health and disease and they are key players at the interface between environmental changes and host biology [19]. A meta-analysis study provide the strong evidence that the efficacy of probiotics is both strain-specific and disease-specific [20]. Various probiotic strains (*Lactobacillus*, *Biofidiobacteria*, *Streptococcus*, *Entrococcus*, *Clostridium*, and *Bacillus*) have different mechanisms-of-action [20]. Several studies have shown that such mentioned endocrine disorders are linked with alteration in gut microbiota diversity and composition [5]. The relation between gut microbiota profile and dietary patterns has also seen [21]. Crucial roles of gut microbiota and probiotics in obesity [22, 23], diabetes [24], NAFLD [10] and metabolic disorders [25] have reported. Probiotics exert their beneficial effect to the host by colonizing in the human body, changing the composition of flora in a certain part of the host and producing active metabolites which can pass through the gut barrier and affect endocrine organs such as liver, pancreas, adipose tissue directly and indirectly and finally maintain healthy condition of host [26]. One of the potential disturbances arising from the intestinal barrier and in microbiota composition change is gut-liver axis dysfunction. Consequences of this event, gut microbiota/ bacterial product and hepatic receptor interactions enhanced and the subsequent events such as oxidative stress, insulin resistance hepatic inflammation, functional and structural changes occurred. Restoring gut microbiota composition, for instance, by probiotic administration, is a therapeutic option to restore induced disorders. Treatment Lep ob/ob mice with combination of bacteria (*Streptococcus, Thermophilus* and several species *of Bifidobacterium* and *Lactobacillus*) ameliorated liver damage, insulin sensitivity, total fatty acid and aminotransferase levels which mainly induced due to decrease of Jun N-terminal kinase (JNK) and NF-kB activation [27]. *Saccharomyces boulardii* Biocodex administration reduced body weight gain and fat mass in obese and type 2 diabetic mice, and significantly changed the gut microbiota composition with an increased proportion of *Bacteroidetes* and a decreased amount of the phyla *Firmicutes*, *Proteobacteria*, and *Tenericutes* [28]. Although number of mechanisms such as anti-inflammation, anti- oxidative stress, and anti-endoplasmic reticulum stress were observed in human and animal in vivo as well as in vitro studies [8, 29]. The particular mechanism of probiotics by which they exert their beneficial effects on endocrine disorders and its related complications are quiet indecisive. One of the main mechanisms (not only mechanism) is modulation of the immune responses via decreasing pro-inflammatory mediators and/ or increasing anti-inflammatory markers among others. Macrophages infiltration into adipose tissue, a source of multipotent adult stem cells, is one of the pathological hallmarks of obesity. Although there are no reports on the influence of intestinal flora on stem cell growth factor-beta (SCGF-beta), a novel protein on obesity. SCGF-β exhibited activity on granulocyte/macrophage progenitor cells in combination with granulocyte-macrophage colony-stimulating factor and macrophage colony-stimulating factor. Obesity-related inflammation causes insulin resistance (IR), which is central to NAFLD or hepatic steatosis. Tarantio et al., showed that prediction of HOMA by measuring SCGF-β levels, possibly mediated by inflammation markers could explain to some extend the inflammatory mechanisms inducing/worsening IR of male patients with obesity-related NAFLD [30]. Another study showed the important role of IL-15 on butyrate-producing bacteria of intestinal compartments and promotes intestinal dysbiosis with butyrate deficiency associated with increased susceptibility to colitis [31]. In fact, age and IL-15 levels were both predictors of early atherosclerosis in a population of obese patients with NAFLD, suggesting a possible role of this cytokine in the atherosclerosis process. Tarantino et al., presented that age and Interleukin-15 levels are independently associated with intima-media thickness in obesity-related NAFLD patients [32].

Normal gut microbiota composition exhibited immunomodulatory function. Alteration in gut microbiota composition was observed in different endocrine disorders. In addition, animals and human investigations confirmed the significant impact of probiotic (gut microbiota modification) on inflammatory mechanisms modulation, however their precise mechanism have not been well known. In this systematic review, we hypothesized that probiotics, through their immunomodulatory effects, could effectively manage endocrine disorders by modulating inflammatory pathways. We investigated various interrelated outcomes so that, their associated effects would support consistent probiotic effects. These objectives were examined by addressing the subsequent questions: [1] what is the effect of probiotics on the outcomes related to obesity, diabetes, NAFLD and metabolic syndrome? [2] What type of inflammatory cytokines or mediators involved in utilizing their beneficial effects? The findings of the present study could have significant therapeutic impact on the management of endocrine disorders.

## Materials and methods

### Focused question

This systematic review was implemented to address “The efficacy of probiotics on endocrinopathies via modulating the inflammatory pathways ˮ.

Eligibility criteria was:

Human and all animal models with experimental endocrine disorders treated with probiotics (all species, all sexes), treatment with probiotic compounds (all timings, frequencies and dosages of treatment), vehicle-treated control human or animals or no treatment, clinical or laboratory manifestation reported, clinical or laboratory manifestation dependent to the disease (e.g., blood glucose, insulin, different blood hormone concentration, and other, these data are assessed by different method such as ELISA which is quantitative test), unrelated outcomes are not reported, English language publications, and focusing on the beneficial effects of probiotic on endocrine disorders via inflammatory mechanisms, published as full manuscripts. Studies which investigating the combination effects of probiotics and other treatment were excluded. We have registered our systematic review in PROSPERO (International prospective register of systematic reviews) and its registration number is: CRD42020213218.

### Search and study selection

We have registered our systematic review in PROSPERO (International prospective register of systematic reviews) and its registration number is: CRD42020213218. Two researchers performed a comprehensive search in the PubMed, and Scopus database in September 2020. The search encompassed all types of articles using the terms included ʻdiabetes̕ OR ʻglucose tolerance̕ OR ʻinsulin sensitivity̕ OR ʻinsulin resistance̕ OR ʻglucagonoma̕ OR ʻmetabolic syndrome̕ OR ʻobesity̕ OR ʻthyroiditis̕ OR ʻhyperthyroidism̕ OR ʻhypothyroidism̕ OR ʻthyroid hormone resistance̕ OR ʻhypopituitarism̕ OR ʻpituitary adenoma̕ OR ʻprolactinoma̕ OR ʻparathyroidism̕ OR ʻosteoporosis̕ OR ʻpolycystic ovarian syndrome̕ OR ʻinfertility̕ OR ʻendocrine disorders̕ AND ʻprobiotics̕ OR ʻnot microbiota̕ OR ʻnot gut microbiota̕. Two researchers independently removed duplicates by hand-screening. Inclusion criteria was the English language publications, and focusing on the beneficial effects of probiotic on endocrine disorders via inflammatory mechanisms. To ensure that the selected articles (based on title and abstract screening by two researchers) meet the inclusion criteria, the full text of articles were also reviewed. After screening the title and abstract, the full text of articles that seemed doubt or relevant were read and non-published in English were omitted. The abstracts not published as full manuscripts, reviews, or the probiotic therapy studies for endocrine diseases other than inflammatory pathways were excluded. No data limitation regarding human or animal, age group, cell type was imposed. Two investigators individually inspected full texts of the potentially eligible articles. Risk of bias was assessed in animal studies using “SYRCLE’s RoB Tool”, and in human investigation using “Cochrane risk-of-bias tool for randomized trials (RoB 2)” guidelines; paper with high bias were omitted.

Data were collected from the full text articles as follows: (i) the type of probiotic, (ii) the endocrine disorder, (iii) the type of study, (iv) inflammatory pathways evaluation used for the assessment beneficial effects of probiotics in treating different endocrine diseases, and (v) the obtained results. Data extraction was done manually by reviewers in our team and no tool were used. Since we had entered the references and we did not use the word inflammation in our search keyword so as not to miss any article. Therefore, we had a large number of articles that needed expertise and high precision to review them, which we could not leave to the software.

Beneficial effects of probiotics on endocrine disorders via inflammatory mechanisms was systematically reviewed and data retrieved from the full text by the authors were included in the manuscript, as the study authors had originally reported (without using any specific or additional analyses). The searches were repeated in June 2021 to identify any new reports that emerged during the time to develop the manuscript but, no new related articles found.

## Results

**Fig. 1:**
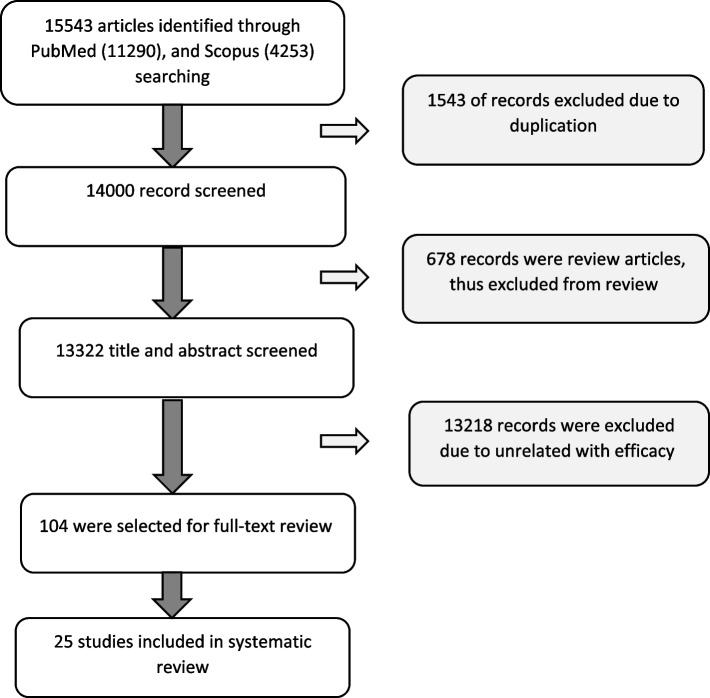
Literature search and study selection flowchart

A total of 15,543 articles was initially identified. After deleting duplications (1543 articles) by two authors using hand- screening, 14,000 papers encountered all inclusion criteria and were selected. Moreover, 653 papers were review articles and omitted (Fig. 1).

Among remained original articles (13322), 13,218 were excluded due to unrelated with topic and 104 articles were selected for full-text review. By studying these articles, only 25 articles included the results of the beneficial effects of probiotic on endocrine disorders via inflammatory mechanisms regulation (Table 1), among them eight articles were related to the evaluation of treatment in human and 17 of them were related to animal models.

**Table 1:** The beneficial effects of probiotic on endocrine disorders via regulation of inflammatory pathways

Probiotics	Endocrine disorder	Animal/ Human	Duration	Key findings	Mechanism(s)	References
Multi-strain probiotics “Symbitter” ^a^	Obesity	Rat	8 Weeks	Probiotic supplementation significantly reduces the prevalence of obesity.	By increasing IL-4 and TGF-β, restoring IL-10, decreasing IL-1β and IL-12Bp40.	Kobyliak, 2018 [33]
Multi-strain probiotics “Symbitter”	Obesity	Rat	3 Months	Probiotics treatment showed significant decreasing of HOMA-IR and rate of obesity.	By decreasing IL-1β, IL-12Bp40 and elevating of TGF-β.	Kobyliak, 2020 [34]
*Lactobacillus mali* APS1 and *L. kefiranofaciens* M1	Obesity	Mice	8 Weeks	Probiotic significantly reduced body weight gain, body fat, liver weight, fat accumulation in the mesenteric adipose and effectively maintained the blood glucose level.	By decreasing TNF-α and IL-6.	Lin, 2016 [35]
VSL#3 probiotic mixture^b^	Obesity	Mice	4 Weeks	Probiotics improved high fat diet-induced steatosis and insulin resistance.	By increasing NKT cell function and number and decreasing TNF-α- IKK-β signaling.	Ma, 2008 [36]
*Lactobacillus plantarum* NCIMB8821	Obesity	Mice	15 Weeks	Probiotics improved glucose homeostasis and metabolic dysfunction	By decreasing MCP-1 and TNF-α and increasing IL-23, IL-33 and TNF-β	Martinic, 2018 [37]
*Enterococcus faecalis* AG5	Obesity	Rat	24 Weeks	Probiotics significantly reduced body weight, BMI, serum cholesterol, triglycerides, improved HDL, insulin and leptin.	By decreasing TNF-α	Mishra, 2020 [38]
*Lactobacillus plantarum* NCIMB8821	Obesity	Mice	10 Weeks	Probiotics prevented the development of insulin resistance, which is at least partly attributable to the prevention of obesity.	By decreasing levels of MCP-1 and IL-6 and TNF-α mRNA	Okubo, 2013 [39]
*Clostridium Butyricum* CGMCC0313	Obesity	Mice	13 Weeks	Probiotics ameliorated obesity, insulin resistance as well as adipose inflammation.	By decreasing TNF-α and increasing IL-10, IL-22 in colon Decreasing TNF-α, IL-6, IL-1β and MCP-A in adipose tissue.	Shang, 2016 [40]
*Bacillus coagulans* *GBI-30608*	Obesity	Mice	5 Weeks	Probiotic reduced food intake, attenuated body weight gain and enhanced glucose tolerance.	By preventing hepatic overexpression of, IL-1β, IL-6.	Urtasun, 2020 [41]
Probiotic yogurt with or without low-calorie diet (LCD)^c^	Obesity	Human	8 Weeks	Probiotics reduced fat percentage, and body weight among overweight and obese individuals.	By reduction in CRP, TNF-α and IL-17	Zarrati, 2014 [42]
Seven strains of lactic-acid-producing bacteria^d^	Diabetes	Mice	12 Weeks	Metformin and probiotics exerted beneficial outcomes on diabetes.	By downregulation of IL-6 and TNF-alpha.	Kattar, 2020 [43]
*Lactobacillus reuteri* GMNL-263	Diabetes	Rat	14 Weeks	Probiotics significantly improved insulin resistance, glucose tolerance, oxidative stress, fatty liver and hepatic damage.	By decreasing IL-6 and TNF-α in concentration adipose tissue**.**	Hsieh, 2013 [44]
Multi-strain probiotics “Symbitter”	Diabetes	Human	8 Weeks	Probiotic therapies modestly improved insulin resistance in patients with type 2 diabetes.	By decreasing IL-1β, IL-6 and TNF-α	Kobyliak, 2018 [45]
*Lactobacillus acidophilus* ZT-L1, *Bifidobacterium bifidum* ZT-B1, *Lactobacillus reuteri* ZT-Lre, and *Lactobacillus fermentum* ZT-L3	Diabetes	Human	12 Weeks	probiotics supplementation had beneficial effects on glycemic control and markers of cardio-metabolic risk.	By reducing CRP	Mafi, 2018 [46]
*L. acidophilus*, *L. bulgaricus*, *L*. *bifidum*, and *L. casei*	Diabetes	Human	6 Weeks	Probiotics reduced insulin resistance.	By decreasing the IL-6 level and increasing CRP	Mazloom, 2013 [47]
“Ecologic®Barrier”^e^	Diabetes	Human	6 Months	Probiotics reduced insulin resistance and concentration of glucose, triglyceride and cholesterol.	By decreasing IL-6, TNF-α and CRP	Sabico, 2019 [48]
*Lactobacillus acidophilus*, *Lactobacillus casei*, *Bifidobacterium bifidum*	Diabetes	Human	12 Weeks	Probiotic supplementation had beneficial effects on glucose homeostasis, and increased insulin sensitivity.	By decreasing CRP	Soleimani, 2017 [49]
*Bifidobacterium longum* DD98	Diabetes	Mice	3 Weeks	Probiotics could alleviate the progression of diabetes.	By decreasing IL-1β, IL-6 and TNF-α	Zhao, 2020 [50]
Multi-strain probiotics^f^	Nonalcoholic fatty liver disease (NAFLD)	Human	1 Year	Probiotic significantly improved liver histology, serum ALT in patients with NAFLD.	By decreasing IL-1β, IL-6, TNF-α and endotoxin in hepatic cells	Duseja, 2019 [10]
*C.* MIYAIRI 588*butyricum* producing probiotic	NAFLD	Rat	8–50 Weeks	MIYAIRI 588 had beneficial effects in the prevention of NAFLD progression	By activating AMPK / AKT/PI3K/Nrf2 pathways and blocking of TNF-α and NF-kB pathways	Endo, 2013 [51]
VSL#3 probiotic mixture	NAFLD	Mice	12 Weeks	Probiotics improved hepatic steatosis.	By decreasing NKT cell activation	Liang, 2014 [52]
Multi-strain probiotics (Lactocare)^g^	NAFLD	Human	8 Weeks	Probiotic supplementation reduced the glycemic indices.	By decreasing TNF-a, and IL-6.	Sepideh, 2016 [53]
*Lactobacillus paracasei* N1115	NAFLD	Mice	16 Weeks	Probiotics were effective in the prevention and treatment of NAFLD.	By repression of lipopolysaccharides, TLR 4 and NF-kB.	Yao, 2019 [54]
Microbiota transplantation	Metabolic syndrome	Mice	16 Weeks	Intestinal microbiota can induce insulin resistance and obesity in an animal model that is genetically protected from these processes.	By activating of TLR4, associated with ER stress and JNK activation	Guadagnini, 2019 [55]
*Lactobacillus rhamnosus* GG	Metabolic syndrome	Mice	12 Weeks	Probiotic treatment may be a potential strategy in the prevention/treatment of metabolic disorders.	Increasing hepatic FGF21 mRNA expression and protein levels, which increased adiponectin production and NF-kB protein level.	Liu, 2020 [56]

^a^Multi- probiotic “symbiter” containing concentrated biomass of 14 probiotic bacteria genera *Bifidobacterium*, *Lactobacillus*, *Lactococcus*, *Propionibacterium*

^b^VSL#3 probiotic mixture consisting of eight bacterial strains (four *Lactobacillus,* three *Bifidobacterium* and one *Streptococcus*)

^c^The probiotic yogurt was made with the strains: *Lactobacillus acidophilus* LA5, *Lactobacillus casei* DN001, and *Bifidobacterium lactis* BB12

^d^Probiotic strains including: *Lactobacillus rhamnosus*, *Saccharomyces boulardii*, *Bifidobacterium breve*, *Bifidobacterium lactis*, *Lactobacillus acidophilus*, *Lactobacillus plantarum*, and *Lactobacillus reuteri*

^e^”Ecologic®Barrier” containing (*Bifidobacterium bifidum* W23, *Bifidobacterium lactis* W52, *Lactobacillus acidophilus* W37, *Lactobacillus brevis* W63, *Lactobacillus casei* W56, *Lactobacillus salivarius* W24, *Lactococcus lactis* W19 and *L. lactis* W58)

^f^It composed of (*Lactobacillus paracasei* DSM 24733, *Lactobacillus plantarum* DSM 24730, *Lactobacillus acidoph- ilus* DSM 24735 and *Lactobacillus delbrueckii* subsp. *bulgaricus* DSM 24734, *Bifidobacterium longum* DSM 24736, *Bifidobacterium infantis* DSM 24737, *Bifidobacterium breve* DSM 24732, and *Streptococcus thermophilus* DSM 24731)

^g^Lactocare containing (*Lactobacillus rhamnosus*, *Lactobacillus casei*, *Lactobacillus acidophilus*, *Bifidobacterium breve*, *Lactobacillus bulgaricus*, *Bifidobacterium longum*, *Streptococcus thermophilus*)

### Predominant probiotics used in treatment of endocrine disorders

Results revealed that therapeutic effects of different probiotics, including *Lactobacillus* species [18], *Bifidobacterium* [12], *Streptococcus* [7], *Enterococcus* [5], *Bacillus coagulans* [5], Lactic acid producing probiotics [5], Butyrate producing probiotic [5] and Multi-strain probiotics [10] were investigated as well as different endocrine disorders including obesity [16], diabetes [14], NAFLD [10] and metabolic syndrome [7]. According to the results, *Lactobacillus* strains were predominantly used for management of endocrine disorders. In obesity and diabetes their mechanisms of action mostly rely on decreasing TNF-α and IL-6 levels. In NAFLD and metabolic syndrome their effects were modulated by repression of lipopolysaccharides, TLR 4 and NF-kB. The mechanism of action of different *Lactobacillus* strains in endocrine disorders has both similarities and differences. The discrepancies in their effects may be due to their difference in genetic levels and also the enzyme and biomolecules that they produce during their growth phase of life. In regard to other probiotic speices, *Streptococcus thermophiles*, *Enterococcus faecalis* AG5, *Bacillus coagulans*, *Clostridium Butyricum* CGMCC0313, and *Bifidiobacteria* have beneficial effects on obesity. For diabetes, *Biofidiobacteria* showed ameliorative effects. In the case of NAFLD, using multi strains of probiotics improved the hepatic steatosis and prevented that condition.

### Probiotic effects on endocrine disorders via modulation of immune system

**Fig. 2:**
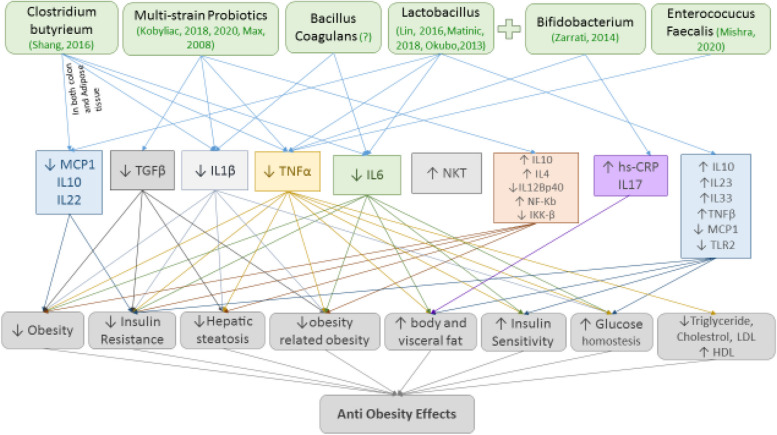
Probiotics ameliorate obesity, decrease insulinresistance, decline hepatic steatosis, increase insulin sensitivity, improve glucose haemostasis, and reduce lipid levels via regulating TGFβ (Transforming growth factor beta), TNFα (Tumor necrosis factor), TLR2 (Toll Like Receptor 2), IL-1 (The Interleukin-1 family), IL-6 (The Interleukin-6), IL-10 (The Interleukin-6), IL-22 (The Interleukin-22), IL-23 (The Interleukin-23), IL-33 (The Interleukin-33), MCP1 (The monocyte chemoattractant protein-1), and NKT (Natural killer T)

**Fig. 3:**
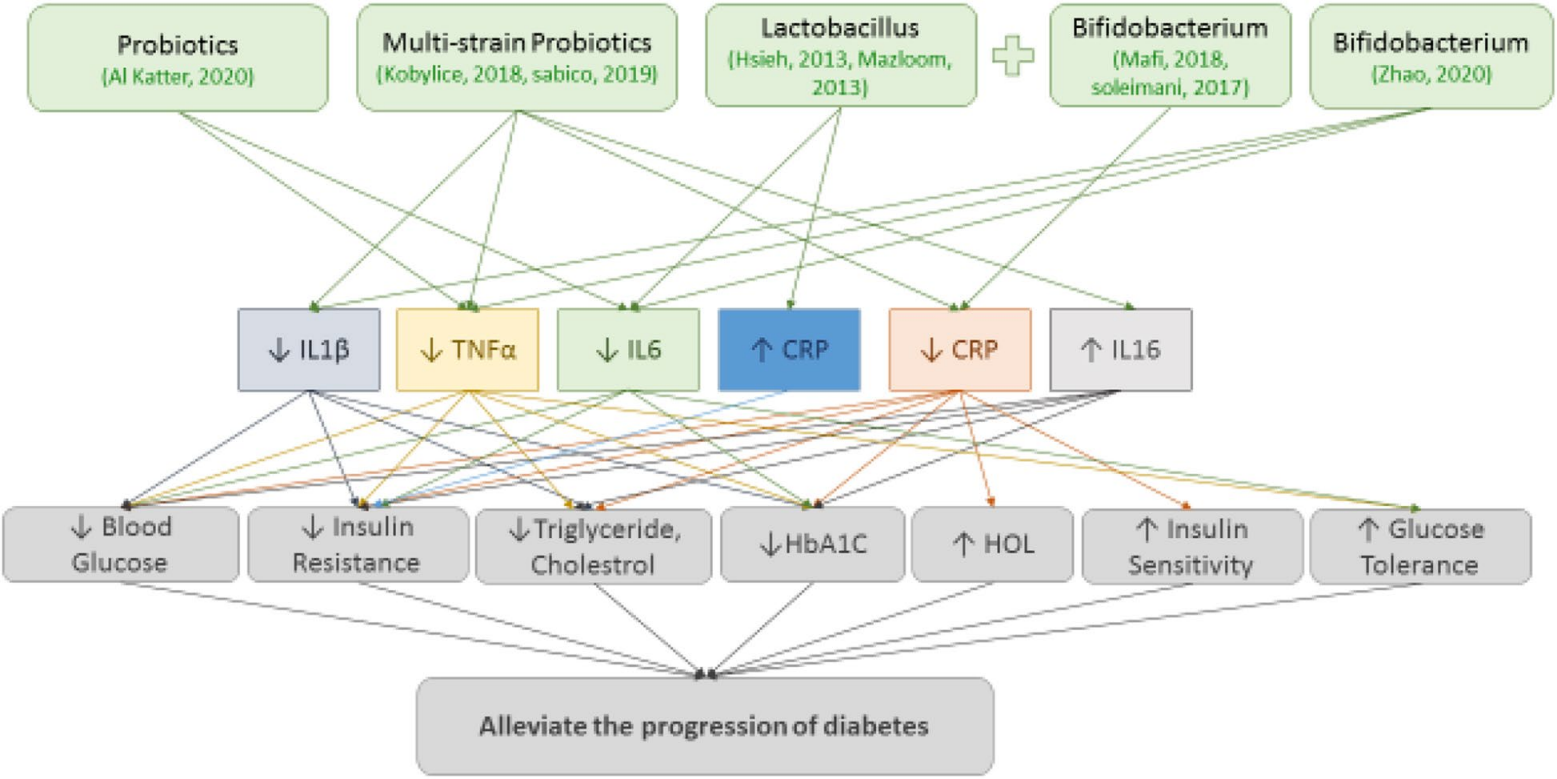
Probiotics alleviate progressing diabetes, decrease HbA1C, enhance insulin sensitivity, improve glucose tolerance via modulating IL-1 (The Interleukin-1 family), IL-6 (The Interleukin-6), CRP (C reactive protein), IL-16 (The Interleukin-16), and TNFα (Tumor necrosis factor)

**Fig. 4:**
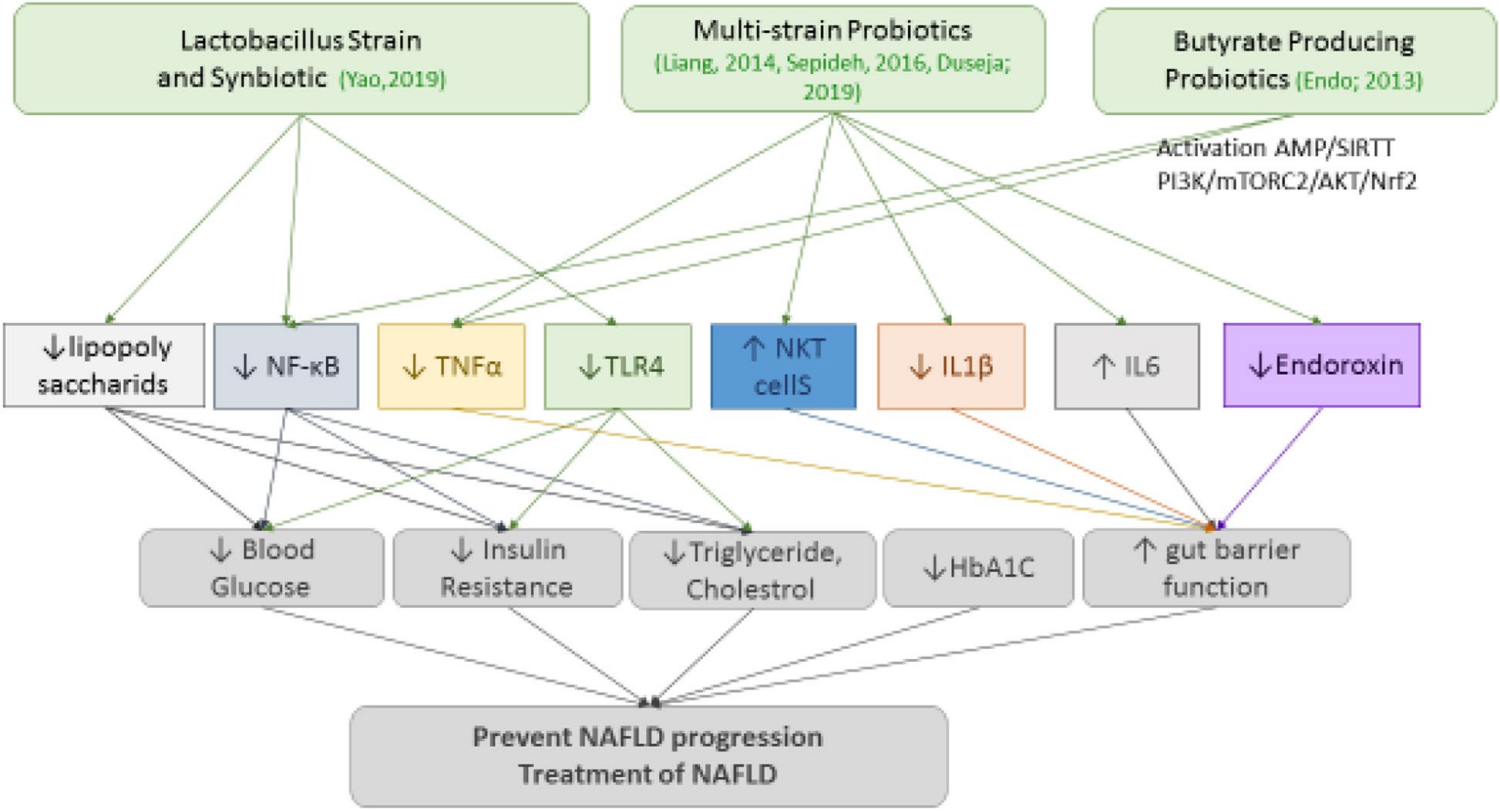
Probiotics prevent NAFLD via maintaining IL-1 (The Interleukin-1 family), IL-6 (The Interleukin-6), TNFα (Tumor necrosis factor), TLR4 (Toll Like Receptor 4), LPS (Lipopolysaccharide), NKT (Natural killer T), NF-κB (Nuclear factor kappa-light-chain-enhancer of activated B cells), and endoroxin

**Fig. 5:**
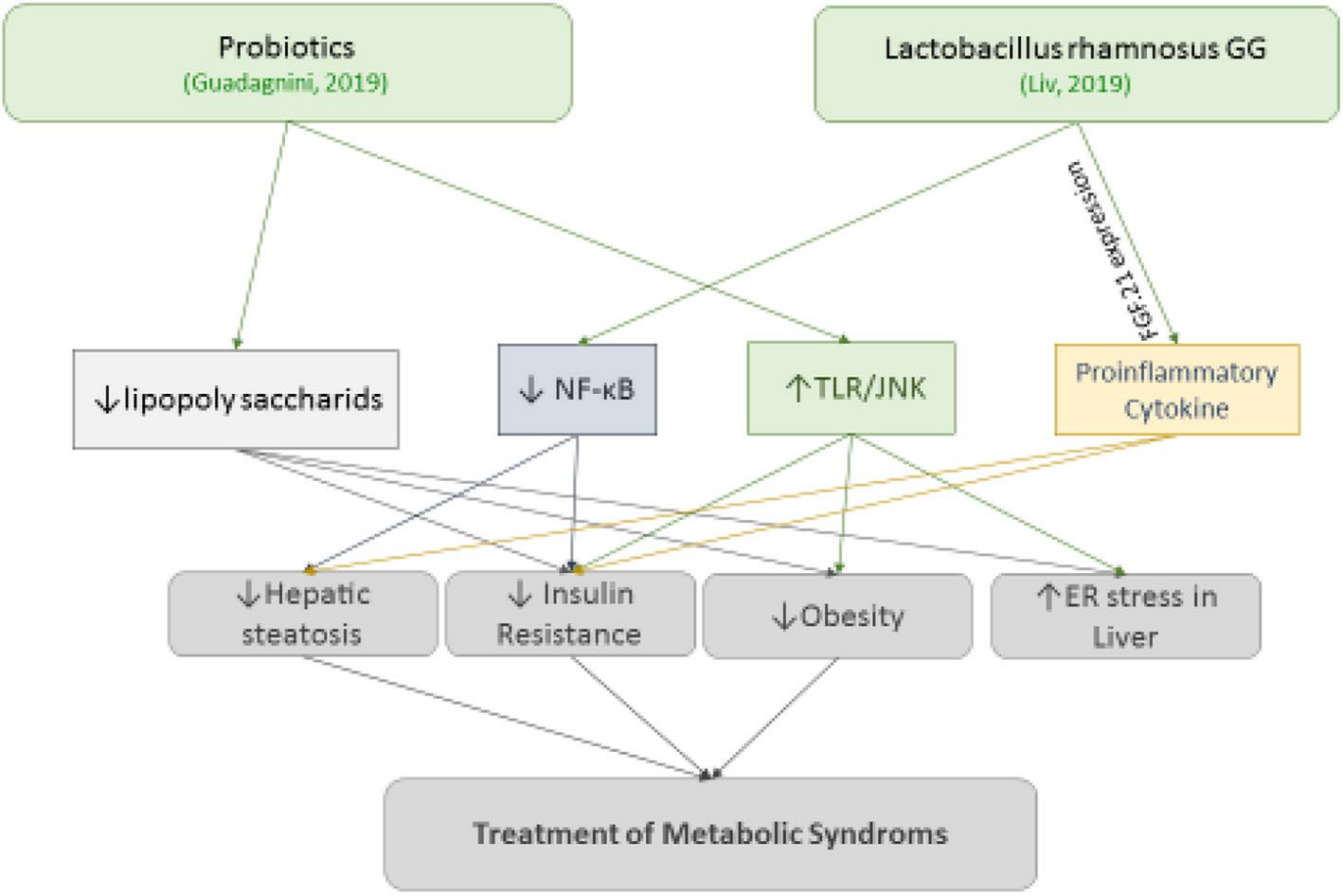
Probiotics manage metabolic syndrome via regulating NF-κB (Nuclear factor kappa-light-chain-enhancer of activated B cells), TLR/JNK (Toll Like Receptor/c-Jun N-terminal kinase), LPS (Lipopolysaccharide) and pro-inflammatory cytokines

Probiotic strains containing (*Lactobacillus*, *Biofidiobacteria*, *Streptococcus*, *Entrococcus*, *Clostridium*, and *Bacillus*) were evaluated from these studies. The inflammatory mechanisms evaluated in these studies included pro-inflammatory cytokine markers (19 studies), anti-inflammatory cytokines (four studies), and other markers or pathways of inflammation (10 studies) in Obesity (Fig. 2), diabetes (Fig. 3), NAFLD (Fig. 4) and metabolic syndrome (Fig. 5). Among these markers, the most studied factor was TNF-α. In the next section, these studies are discussed based on the investigated inflammatory mechanism as well as the endocrine disorders. Each endocrine disorder might possess numerous perilous indicators, yet Probiotic solely influences a range of such indicators, and we have incorporated exclusively those impactful indicators to prevent unnecessary effort.

## Suppression of pro-inflammatory markers

### Interleukin-1β

Seven studies with patients or animals using different type of probiotics such as multi strain [10, 33, 34], *Clostridium butyricum* CG MCC 0313.1 [40], *Bacillus coagulans* [41] and *Biofidobacterium longum* DD28 displayed the role of IL-1β in different endocrine disorders. Their results indicated that probiotics could significantly prevent or lower the rate of obesity, protect animals against the development of obesity, improve obesity related insulin resistance [40], ameliorate insulin resistance [45], prevent NAFLD progression [10] and hepatic steatosis [41], and alleviate the progression of diabetes [28] that these effects were mediated partly via decreasing IL-1β.

### Interleukin-6

Nine studies examined changes in IL-6 by using various probiotics in different endocrine disorders [40, 41, 44, 45, 48, 50, 53, 57] (Table 1). Administration of probiotics like *Lactobacillus* [44, 57], *Bacillus coagulans* [41], *Bifidobacterium longum* [50] and Multi strain probiotics [(*Lactobacillus, Bifidobacterium, Propinobacterium, Acetobacter genera*) and (*Lactobacillus casei, L. acidophilus*, *L. rhamnosus*, *L. bulgaricus*, *Bifidobacterium lingum*, *B. brev*, *Streptococcus thermophiles*)] [45, 53] exerted therapeutic effects on diabetes [43, 44, 50], improve insulin resistance [40, 43, 45, 48], prevent hepatic steatosis [41, 53] and protect from obesity [40] in human or animal with endocrine disorders. These beneficial effects were partly associated with decreasing IL-6 levels.

### Interleukin-12

The use of Multi strain probiotics (*Lactobacillus, Bifidobacterium, Propinobacterium, Acetobacter genera*) and (*Bifidobacterium* VLK, *Bifidobacterium* VKB, *Lactobacillus casei* IMVB-7280) resulted in a significant decrease in the level of pro- inflammatory cytokines such as IL-12Bp and decreasing the rate of obesity in rats [33, 34].

### Tumor necrosis factor-𝛼

Thirteen studies reported the impact of different type of probiotics on TNF-𝛼 in human or animals with endocrine disorders. Probiotic supplementation via activating AMPK/SIRT1/PI3K/mTORc2/AKT/Nrf2 pathways resulting in blocking TNF- 𝛼 [51] or decreasing its level [10, 43, 53], could prevent the progression of diabetes or NAFLD. Results of other studies disclosed that probiotic administration through suppressing or decreasing TNF- 𝛼 exerted therapeutic effects such as improving insulin resistance in diabetic patients or animals [44, 45, 48]. Probiotic treatment effectively prevents obesity or improve obesity-related insulin resistance which was partly associated with reducing TNF-𝛼 levels [36, 39, 40].

### Tumor necrosis factor-β

Only a single study reported that the *Lactobacillus plantarum* NCIMB8821 administration could improve markers of metabolic dysfunction in obese mice. These beneficial effects were partly mediated via increasing the level of TNF- β [38].

### NF-kβ

The influence of probiotics on NF-kβ was evaluated in three studies [37, 43, 54] and using a different type of probiotics exhibited beneficial effects on prevention and progression of NAFLD [51, 54] and metabolic syndrome [56] that was associated with decreasing of NF-kβ protein level.

### MCP-1

The use of *Clostridium butyricum* CGMCC0313.1*, Lactobacillus plantarum* NCIMB8821 and *Clostridium butyricum* resulted in a significant decrease in the MCP-1 levels, which led to improved metabolic dysfunction, prevention of obesity and improvement of obesity -related insulin resistance in animals [37, 39, 40].

## Enhancing anti-inflammatory markers

### Interleukin-4

Just one study examined IL-4 changes after the use of probiotic in obese rats [33]; in this study, the IL-4 level was significantly increased and consequently, reduced the prevalence of obesity in rats after using Multi-strain probiotics.

### Interleukin-10

Only Two studies had reported that intestinal bacteria modulation by *Lactobacillus* or Multi-strain probiotic administration could treat obesity, which was partly mediated via upregulating the production of IL-10 [33, 57].

### Interleukin-22

Shang et al., exhibited that butyrate-producing probiotic *Clostridium butyricum* CGMCC0313.1 (CB0313.1) administration could significantly enhance IL-22 level in colon tissue of obese mice [40].

### Interleukin-23 and Interleukin-33

Martinic et al., in 2018 reported that supplementation with *Lactobacillus plantarum* NCIMB8821 could significantly increase anti-inflammatory cytokines, including IL-23 and IL-33 resulted in improving markers of metabolic dysfunction in obese mice [39].

### Transforming growth factor-β

Use of Multi-strain probiotics (*Bifdobacterium* VLK*, Bifidobacterium* VKB*, Lactobacillus casei* IMVB-7280) with or without nutraceutical supplementation led to significant increasing in serum TGF- β level and consequently reduced remarkably the prevalence of obesity in animals [33].

## Effects on other inflammatory markers

### C-reactive protein

Data concerning the impact of probiotics on CRP levels of patients or animals with endocrine disorders were extracted from five articles. Probiotics supplementation including Multi strain probiotics (*Bifidobacterium bifidum* W23, *Bifidobacterium lactis* W52, *Lactobacillus acidophilus* W37, *Lactobacillus brevis* W63, *Lactobacillus casei* W56, *Lactobacillus salivarius* W24, *Lactococcus lactis* W19 and *L. lactis* W58) [48], or probiotic containing *L. acidophilus, L. casei, and B. bifidum* [49], or containing *Lactobacillus acidophilus La5, Bifidobacterium BB12*, and *Lactobacillus casei DN001* [42], or probiotic supplements containing *Lactobacillus acidophilus* strain ZT-L1, *Bifidobacterium bifidum* strain ZT-B1, *Lactobacillus reuteri* strain ZT-Lre, and *Lactobacillus fermentum* strain ZT-L3 [46], could reduce CRP levels and subsequently resulted in exerting positive effects on glycemic control, and cardio- metabolic risk in diabetic patient and also decreased fat percentage and body weight in overweight and obese individuals. While, administration probiotic containing *L. acidophilus*, *B. longum*, and *L. casei* cause to non-significant increase of c-reactive protein concentration in diabetic patients for which the authors did not mention a justification.

### Toll like receptors

The effects of probiotic via alteration in TLRs are reported in two studies. Guadagnini et al., in their study presented that probiotic transplantation by changing in gut microbiota and integrity of intestinal barrier increased lipopolysaccharides and through an increase in TLR/JNK pathway in the liver caused an elevation in ER stress and downregulating insulin signaling in TLR2^−^/^−^ mice [55]. *Lactobacillus paracasei* administration could effectively prevent and treat NAFLD that was mediated by transcriptional suppression of inflammatory factors such as TLR_−_ 4 [54].

### Lipopolysaccharides

One study reported that intestinal bacteria modulation by probiotic administration via repression of lipopolysaccharides and consequent suppression of TLR-4 and NF-kB pathways could prevent or treat NAFLD [56]. The results of the other study exhibited that probiotic intake in TLR2^−^/^−^ mice by increasing lipopolysaccharides and then TLR/JNK pathway activation resulted in downregulating insulin signaling pathway and inducing insulin resistance in the liver and muscle tissue of animals [55].

### Natural killer T cells

Intestinal bacteria alteration by using single or multi strain probiotics could directly increase NKT cell function [36, 52] in both in vivo and in vitro condition as well as NKT cell number [36] and can be used as a therapeutic option for treating NAFLD [52] and obesity related diseases [51].

## Discussion

Results of this systematic review indicated that distinct Lactobacillus strains were the most investigated probiotics in obesity, diabetes and metabolic syndrome as well as multi strain probiotics in NAFLD. Also, TNF-α was the most examined inflammatory factor (Pro-inflammatory) in obesity and NAFLD, and IL-6 was the most explored pro-inflammatory factor in diabetes. These findings confirmed our hypothesis and demonstrated that modulation of inflammatory pathways is effective mechanisms in inducing beneficial effects of probiotics in treating different endocrine diseases.

The endocrine system is an integrated network regulating many internal body functions through hormone secretion. So, endocrine system dysfunction can lead to many disorders throughout the body. Therefore, their treatment is crucial for hampering or reducing difficulties caused by an endocrine system imbalance such as insufficient insulin secretion or response [7] and insulin resistance in obesity [8, 9] or NAFLD [10].

Several studies have shown that such mentioned endocrine disorders are linked with alteration in gut microbiota diversity and composition [5]. Both gut microbiota and probiotics have a pivotal role in endocrine debases such as obesity [22, 23], diabetes [24], NAFLD [10] and metabolic disorders [25] have been reported. Although number of mechanisms such as anti-inflammation, anti- oxidative stress, and anti-endoplasmic reticulum stress were observed in human and animal in vivo as well as in vitro studies [8], the particular mechanism of improvement effects of probiotics on endocrine disorders are quiet indecisive. One of the main mechanisms (not only mechanism) is modulation of the immune responses via decreasing pro-inflammatory mediators and/ or increasing anti-inflammatory markers among others. This systematic review revealed that probiotic consumption improves different endocrine disorders via modulating of the immune responses, which is discussed below in this regard.

Obesity as a common global health problem [58, 59], affects millions of people worldwide. According to the data of the World Health Organization, over 600 million humans have obesity [60]. Obesity rates are estimated to double by 2030. Several factors such as genetic susceptibility, environmental conditions, human lifestyle and variations in the diversity and abundance of the microbiota are contributing to obesity [9]. On the other hand, long term consumption of high fat diet in mice significantly led to the change normal probiotic composition of colon. For instance, it reduced *Bifidobacterium* and *Lactobacillus* concentrations [9].

Crucial roles of gut microbiota and probiotics in obesity [22, 23] have been reported. In the recent years, increasing attention has been given from the scientific community to the experimental and clinical studies supporting the role of probiotics in the management or treatment of obesity.

Overall, probiotic administration revealed anti-obesity effects and the metabolic status of obese subjects or animals improved as indicated by reducing body weight (BW) [38, 41, 57], body mass index (BMI) [38, 42], body fat mass (BFM) [42, 57], Visceral fat [57], mesenteric adipose tissue weight [37], and lipid deposition in the liver [45]. Probiotics also decrease insulin resistance [34, 36, 37, 40], reduce triglyceride, cholesterol and LDL concentration [38], improve insulin sensitivity [34] and glucose homeostasis [34, 39] and also lower hepatic steatosis [36, 41]. One of the underlying mechanism of these beneficial effects is modulating inflammatory pathways, including declining pro-inflammatory cytokines such as TNF-α [36, 37, 42, 57], IL-6 [40, 41, 57], IL-1β [34, 41], IL-12Bp [33, 34] or increase anti- inflammatory cytokines like IL-4 [33], IL22 [40], IL-23 [39], IL-33 [39], TGFβ [33, 34] and IL-10 [33, 57] or change in other inflammatory markers such as IL-17 [42], IKK-β [33], MCP-1 [37, 39, 40] and CRP [42]. It should be noted mentioned that, unlike other pro-inflammatory factors which their reduction induces ameliorating or protective effects in obesity, the increase of pro-inflammatory factor, TNF-β leads to improving effects in obesity [40, 41, 45]. The effects of TNF-β on GI and mucosal integrity was shown, so increasing levels of this pro-inflammatory cytokine is partly responsible to inducing therapeutic effects in obesity [38].

High-fat diet caused to hepatic steatosis and insulin resistance mediated by NKT cell depletion. Probiotic administration could restore NKT cells and consequently restore high-fat diet-induced metabolic disorders in mice [36]. Drawing upon the findings of previous studies, therapeutic approaches targeting gut microbiota adjustment specially which one inducing inflammatory pathways modulation would be regarded as potential effective treatment to obesity and consequently decrease the prevalence of obesity and obese population through the world.

Diabetes mellitus is the other most commonly endocrine diseases occurring and rapidly growing comorbid [43]. WHO estimates that, globally 592 million adults will be living with diabetes in 2035 [61]. Probiotics intake improved glucose control and tolerance [43, 44, 46, 48–50], decrease insulin resistance [44–48], increase insulin sensitivity [46, 49], diminish HbA_1_c [40, 49, 50], reduce cardio- metabolic risks such as declining TG and cholesterol, rising HDL concentration [46, 48] and decreasing diabetic associated damages in the liver [44, 50] and pancreas [44] that generally alleviated the progression of diabetes. These improving impacts were partly due to inflammatory pathway modulation. In consistent with obtained results regarding reduction of pre-inflammatory changes in obesity, suppressing an decreasing of TNF-α and IL-6 production in adipose tissue or serum [43–45, 47, 48], reducing IL-β1 [45, 50]. There are conflict results in regard to CRP levels, anti- inflammatory cytokine, in diabetes [46–49]. The improving effect was induced partly due to decreasing levels of CRP [30, 57] while, in the other investigations these effects were induced by increasing CRP levels (42,44). Consumption yogurt containing probiotic such as *Lactobacillus acidophilus* strain ZT-L1, *Bifidobacterium bifidum* strain ZT-B1, *Lactobacillus reuteri* strain ZT-Lre, and *Lactobacillus fermentum* strain ZT-L3 increased CRP level and exhibit positive impacts in diabetic patients such as remaining blood glucose level in normal range. The reduction of the risk of cardio metabolic disorders observed in these patients is also due to the normal glycaemia caused by the increase in CRP level [43]. Consistent with obtained results of these studies probiotics by modulating inflammatory pathways are proposed as considerable treatment option of diabetes which occurs partly due to activation of inflammatory signaling. It should be noted that one of the main advantages of this method is that probiotics are compatible biomedicine because the presence of gut microbiota.

Nonalcoholic fatty liver disease (NAFLD), is currently the most frequent cause of chronic liver disease, becoming a serious health concern that threatens the well-being of a significant number of people across the world. Several factors such as obesity, unhealthy dietary patterns and sedentary lifestyles are contributing to create NAFLD [62]. A number of studies have described the advantageous effects of probiotics in NAFLD that were linked to modulated inflammatory responses. In line with observed results in obesity and diabetes, probiotic intake through elevating hepatic NKT cells function [52] and decreasing the level of pro-inflammatory cytokines: TNF-α, IL-6 and IL-1β [10, 53] and endotoxins in hepatic cells could significantly improve hepatic steatosis, liver histology and function [10, 53], progress glycemic indices and decrease insulin resistance [53] in both human and animal models of NAFLD. Endo et al. have reported that 8–16 weeks’ administration of *C. butyricum* MIYAIRI 588 (butyrate-producing probiotic) to mice with NAFLD significantly decreased gut derived endotoxin levels in the portal blood by changing the intestinal flora and restoring gut-barrier functions, as well as lowering the levels of pro-inflammatory cytokine TNF-α in liver which results in regulating transcription factor NF-kB thereby causing inhibition of NAFLD progression [51]. The other study displayed that *Lactobacillus paracasei* N1115 dietary supplementation diminished serum total triglyceride and cholesterol, decrease the fasting blood glucose and insulin and can effectively prevent and treat NAFLD in an experimental model that was partly associated with repression of inflammatory factors such as lipopolysaccharides, TLR4 and NF-kB [54]. In line with Yao F et al. study, Guadagnini et al. presented that in TLR^−^/^−^ mice intestinal epithelial barrier integrity was impaired, gut microbiota composition was changed, and blood levels of lipopolysaccharides was enhanced which results in glucose intolerance, and body weight gain. Probiotic treatment of these animals via activation of TLR4/JNK pathways and increase ER stress in liver caused to insulin signaling downregulation in both liver and muscle tissue, however not affect activation of NF-ƙB pathway [55]. Administration different dose of VSL#3 or *bifidobacterium infantid* to high-fat diet-induced NAFLD increased the number of NKT cells in hepatic cells in both in vitro and in vivo condition, and restore hepatic steatosis. This effectswere dose- and probiotic strain-dependent, high dose of VSL#3 was more effective. In other word, hepatic NKT cells adjustment is followed by probiotics administration and gut microbiota alteration, and eventually restore hepatic steatosis. Although the precise role of NKT cells in the pathogenesis of NAFLD is debatable, it seems that NKT cells play a significant role in regulating immune responses in hepatocytes in NAFLD [36].

Metabolic dysfunction are affected by genetics and environmental. The role of gut microbiota in inducing metabolic dysfunction has been proven, gut microbiota itself is influenced by genetic and environmental factors. One of the regulator of the intestinal microbiota is immune system, and among different molecules of this system the pivotal role of TLRs has been confirmed. Results exhibited that probiotic intake in TLR2−/− mice via enhancing lipopolysaccharides concentration, TLR/JNK pathway activation, ER stress, and downregulation of insulin signaling in both in both liver and muscle tissue [55] and increasing FGF-21 expression that led to activation of butyrate-mediated PPARα, enhance of adipose tissue adiponectin expression could improve metabolic disorders and prevent obesity, insulin resistance and hepatic steatosis in mice with metabolic syndrome [55, 56]. As mentioned above compelling evidence indicate that gut dysbiosis plays a vital role in developing metabolic syndrome [9], combination of endocrine disorders, and probiotic supplementation has been used as a new approach to prevent or treat metabolic dysfunction, and this has gained remarkable attention in recent years [56].

Based on this review, there is limited clinical evidence to confirm the positive effects of probiotic on inflammatory response modulation for better management of endocrine disorders. Clinical trials involving the therapeutic use of probiotic supplementation have yielded less than impressive results [63, 64]. In addition, there is a lack of assessment and systematic reporting of adverse events in probiotic intervention studies, and interventions are poorly documented. Although published available evidence regarding the safety of probiotic have not reported any increase risk of probiotic application, they may cause adverse effect in some patient whom receiving radiotherapy [65] which is a gap and therefore investigating the effects of probiotics on endocrine disorders through the modulation of inflammatory pathways in the presence of other diseases is suggested in future studies.

There is a significant potential for preventing or treating human disease through the microbiome. Currently, there are numerous disease-microbiome connections documented in literature, but the successful utilization of these connections remains limited. One possible reason for the limited translation of microbiome science into microbiome medicine is the considerable variability observed in different studies. While a specific enterotype or microbial species might exhibit positive effects, their effectiveness cannot be generalized to all cases [66]. Conducting more well designed clinical trials with good sample size and also intervention using targeted probiotic strains could be helpful in filling this gap between microbiome and disease in future studies.

This systematic review had some limitations. One of the limitation of the study is publication bias. In this systematic review we considered literature published in English. Furthermore, due to the limited original data, we were unable to evaluate other inflammatory cytokines and other probiotic strains. Only major endocrine disorders including diabetes, obesity, NAFLD, and metabolic syndrome were investigated.

## Conclusion

Results of this systematic review indicated that distinct Lactobacillus strains were the most investigated probiotics in obesity, diabetes and metabolic syndrome as well as multi strain probiotics in NAFLD. Also, TNF-α was the most examined inflammatory factor (Pro-inflammatory) in obesity and NAFLD, and IL-6 was the most explored pro-inflammatory factor in diabetes. These findings confirmed our hypothesis and demonstrated that modulation of inflammatory pathways is effective mechanisms in inducing beneficial effects of probiotics in treating different endocrine diseases. However, the reasons for using different probiotics in different endocrine disorders and the reasons for targeting specific inflammatory cytokines in treatment different endocrine disorders are not mentioned in the reviewed articles. This issue can be the subject of future studies. It seems that the variation in the choice of pro-inflammatory factors across different endocrine disorders is based on the most effective cytokines in each disorder and the relation between each cytokine and pathogenesis of specific diseases. For example, the significance of TNF-α in obesity and NAFLD, as well as IL-6 in diabetes has been reported. TNF-α is overexpressed in and secreted by adipose tissue of obese animals and humans, and its levels correlate to the degree of adiposity and insulin resistance [67]. Experimental evidence suggests that TNF-α is a cytokine with a critical role in the pathogenesis of NAFLD. Although, the production of TNF-α may be an early event during the course of nonalcoholic fatty liver (NAFL), TNF-α may play a more substantial role in the pathogenesis of nonalcoholic steatohepatitis (NASH) and NAFLD-associated fibrosis [68].

A vast number of epidemiological, genetic, rodent, and human in vivo and in vitro studies have investigated the putative role of action/lack of action of IL-6 in the pathogeneses underlying obesity, insulin resistance, β-cell destruction, type 1 diabetes, and type 2 diabetes. These studies suggest both protective and pathogenetic actions of IL-6 in diabetes. IL-6 induces insulin resistance in adipose tissue and liver and may synergize with proinflammatory cytokines to produce β-cell damage [69].

There is little evidence about the beneficial effects of probiotics via inflammatory mechanisms in the treatment or prevention of endocrine disorders. Also, there is no similar probiotics regimen, several different strains and amount of probiotics, and different treatment duration was assessed in different studies. So, more studies are required to conclude the importance of inflammatory factors in exerting beneficial effects of probiotic in different cells and tissues.

Distinct *Lactobacillus* strains were the most investigated probiotics in obesity, diabetes and metabolic syndrome as well as multi strain probiotics in NAFLD. Also, TNF-α was the most examined inflammatory factor (Pro-inflammatory) in obesity and NAFLD, and IL-6 was the most explored pro-inflammatory factor in diabetes.

The long-term genetic stability, the antibiotic susceptibility and translocation rate of *Lactobacillus* strains may be the reason for it predominant use in the most studies. Furthermore, experimental and clinical evidence supports effectiveness of *lactobacilli* for treatment of several pathological conditions. Long-term consumption of *lactobacilli* induces qualitative and quantitative modifications in the human gastrointestinal microbial ecosystem.

There is little evidence about the beneficial effects of probiotics via inflammatory mechanisms in the treatment or prevention of endocrine disorders. Also, there is no similar probiotics regimen, several different strains and amount of probiotics, and different treatment duration was assessed in different studies. So, more studies are required to conclude the importance of inflammatory factors in exerting beneficial effects of probiotic in different cells and tissues. The results of this review proposed designing future studies to investigate the effects of specific probiotic strain in one of the endocrine disorder or investigate effects of specific probiotic strain in one of the endocrine disorder via alteration of specific inflammatory factor. Moreover, investigating the effects of probiotics on endocrine disorders through the modulation of inflammatory pathways in the presence of other diseases is suggested in future studies.

## Data Availability

No datasets were generated or analysed during the current study.

## Abbreviations

AKT: protein kinase B
AMPK: AMP- activated protein kinase
BFM: body fat mass
BMI: body mass index
BW: body weight
CRP: C- reactive protein
FGF-21: fibroblast growth factor-21
HDL: high density lipoprotein
IKK-β: inhibitor of nuclear factor B kinase subunit beta
IL: Interleukin
JNK: c-Jun N- terminal kinases
LDL: low density lipoprotein
MCP: monocyte cytokine chemoattractant protein
mTORc2: mammalian target of rapamycin complex 1
NAFLD: non-alcoholic fatty liver disease
NF-kB: nuclear factor kappa-light-chain-enhancer of activated B cells
NKT cell: natural killer T cells
Nrf2: nuclear factor erythroid 2- related factor 2
PI3K: phosphoinositide 3- kinase
SIRT1: Sirtuin
TG: triglyceride
TGF-β: transforming growth factor-β
TLRs: toll like receptors
TNF-α: tumor necrosis factor- α
TNF- β: tumor necrosis factor-β
WHO: world health organization
